# Crystal structure of (μ-*N*,*N*′-di­benzyl­dithio­oxamidato-κ*N*,*S*:*N*′,*S*′)bis­[(η^3^-crotyl)palladium(II)]

**DOI:** 10.1107/S2056989015001292

**Published:** 2015-01-28

**Authors:** Giuseppe Bruno, Santo Lanza, Antonino Giannetto, Alessandro Sacca, Hadi Amiri Rudbari

**Affiliations:** a Department of Chemical Sciences, University of Messina, Via F. Stagno d’Alcontres 31, 98166 Messina, Italy; b Faculty of Chemistry, University of Isfahan, Isfahan, Iran

**Keywords:** crystal structure, dinuclear palladium(II) complex, di­benzyl­dithio­oxamidate, crot­yl

## Abstract

In the centrosymmetric dinuclear title compound, [Pd_2_(C_4_H_7_)_2_(C_16_H_14_N_2_S_2_)], the metal atom is η^3^-coordinated by three C atoms of a crotyl ligand [Pd—C = 2.147 (4), 2.079 (5) and 2.098 (5) Å], the longest distance influenced by the steric inter­action with the benzyl substituents of the di­benzyl­dithio­oximidate (DTO) ligand. The Pd—N and Pd—S bonds to this ligand are 2.080 (3) and 2.3148 (9) Å, respectively, completing a square-planar coordination environment for Pd^II^. The benzyl groups are oriented so as to maximize the inter­action between a benzylic H atom and an S atom, resulting in a dihedral angle of 77.1 (2)° between the benzene rings and the metal complex plane. In the crystal, no inter-complex hydrogen-bonding inter­actions are present.

## Related literature   

For background to structures similar to that of the title compound in which Pd^II^ atoms are linked to allyl groups, see: Lanza *et al.* (2003 ▸, 2011 ▸). For the stereochemical descriptor of a η^3^-crotyl plane, see: Schlögl (1967 ▸). For stereochemical descriptors of a palladium square plane, see: Lanza *et al.* (2000 ▸). For the chemistry of (η^3^-all­yl)palladium, see: Jalòn *et al.* (2005 ▸).
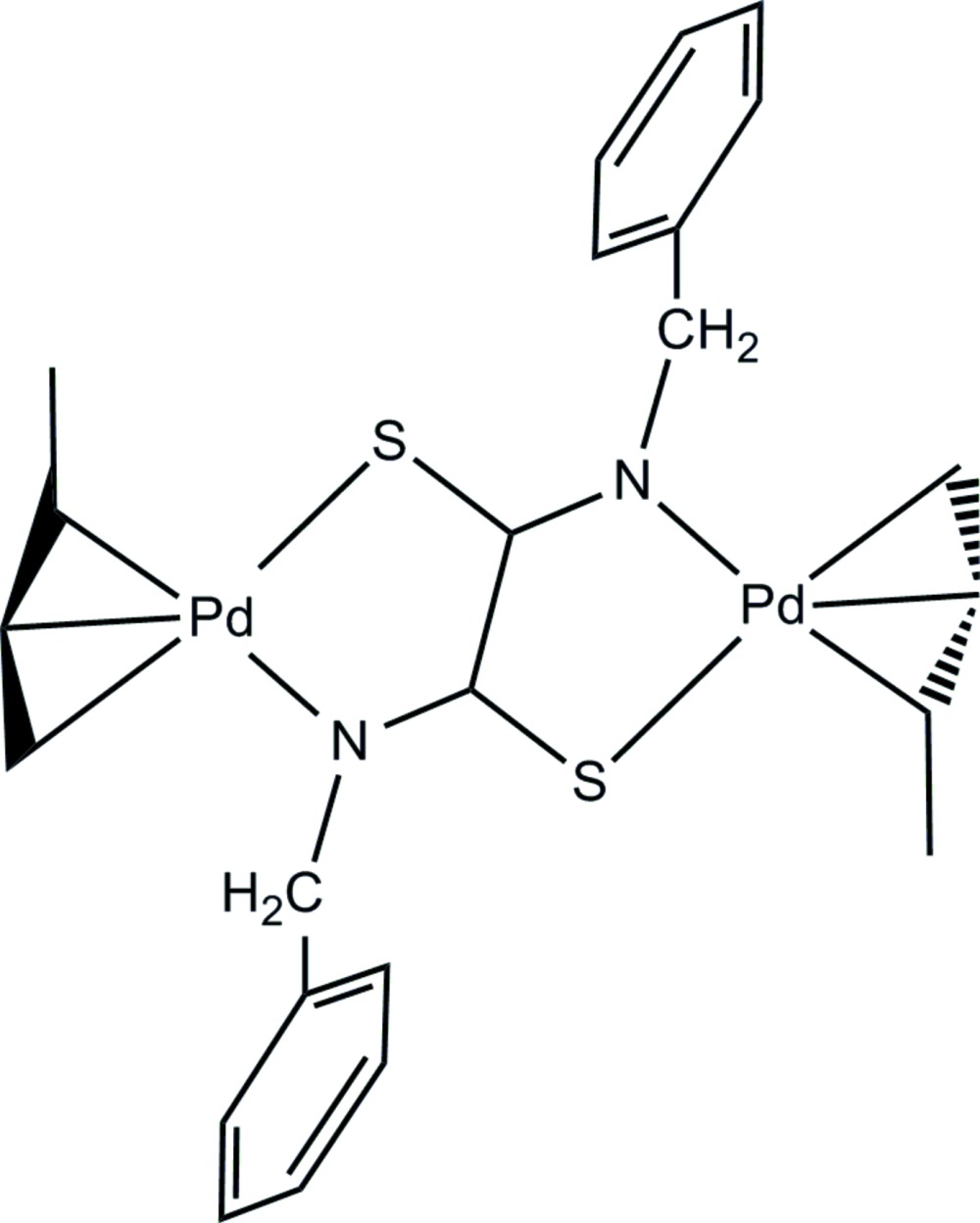


## Experimental   

### Crystal data  

[Pd_2_(C_4_H_7_)_2_(C_16_H_14_N_2_S_2_)]
*M* *_r_* = 621.40
Monoclinic, 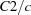

*a* = 18.3240 (2) Å
*b* = 7.1660 (1) Å
*c* = 19.5080 (2) Å
β = 109.341 (4)°
*V* = 2417.03 (7) Å^3^
*Z* = 4
Mo *K*α radiation
μ = 1.67 mm^−1^
*T* = 298 K
0.35 × 0.10 × 0.08 mm

### Data collection  

Bruker APEXII CCD diffractometer
Absorption correction: multi-scan (*SADABS*; Bruker, 2012 ▸) *T*_min_ = 0.611, *T*_max_ = 0.746
34315 measured reflections
2638 independent reflections
2474 reflections with *I* > 2σ(*I*)
*R*_int_ = 0.019

### Refinement  

*R*[*F* ^2^ > 2σ(*F* ^2^)] = 0.031
*wR*(*F* ^2^) = 0.085
*S* = 1.03
2638 reflections
136 parameters
H-atom parameters constrained
Δρ_max_ = 1.36 e Å^−3^
Δρ_min_ = −0.62 e Å^−3^

### 

Data collection: *APEX2* (Bruker, 2012 ▸); cell refinement: *SAINT* (Bruker, 2012 ▸); data reduction: *SAINT*; program(s) used to solve structure: *SHELXS97* (Sheldrick, 2008 ▸); program(s) used to refine structure: *SHELXL97* (Sheldrick, 2015 ▸); molecular graphics: *SHELXTL* (Sheldrick, 2008 ▸); software used to prepare material for publication: *SHELXTL*, *PLATON* (Spek, 2009 ▸) and *enCIFer* (Allen *et al.*, 2004 ▸).

## Supplementary Material

Crystal structure: contains datablock(s) global, I. DOI: 10.1107/S2056989015001292/zs2324sup1.cif

**Fig. 1.**
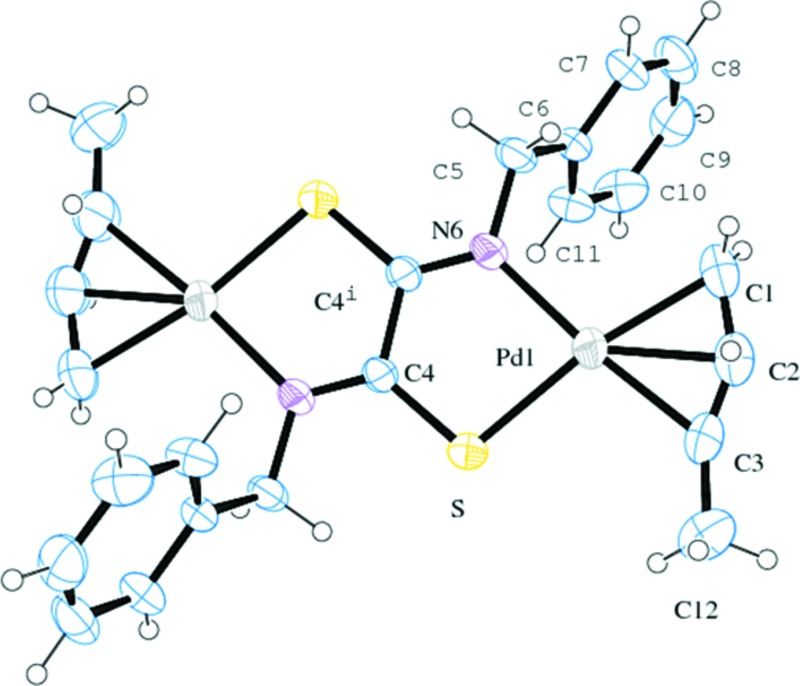
A perspective view of the centrosymetric title complex, with atom numbering and non H-atoms represented as 40% probability displacement ellipsoids. Symmetry code (i): -x, -y, -z + 1.

**Fig. 2.**
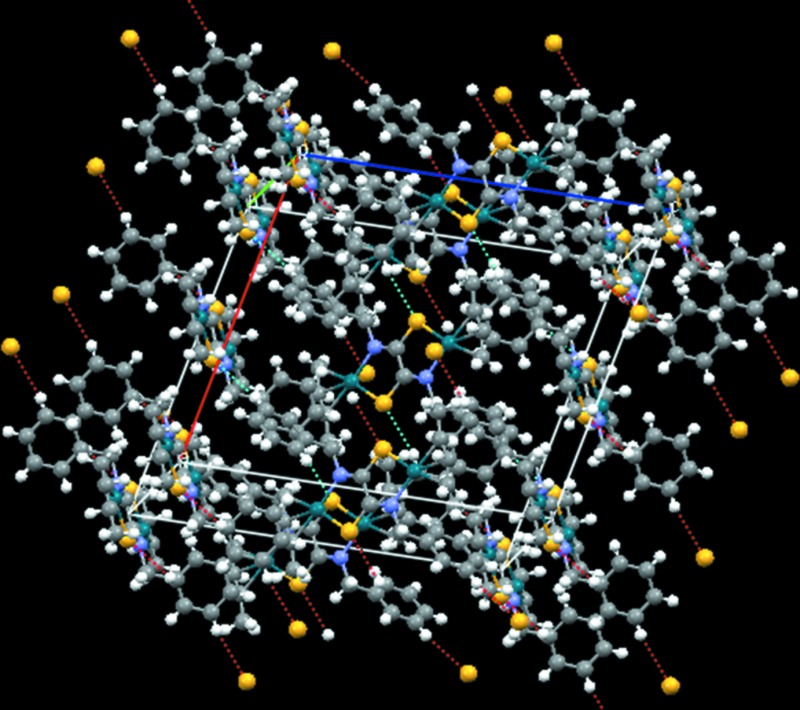
A packing diagram of the title complex viewed along the b axis with C—H···S interactions shown by dotted lines.

Structure factors: contains datablock(s) I. DOI: 10.1107/S2056989015001292/zs2324Isup2.hkl

x y z . DOI: 10.1107/S2056989015001292/zs2324fig1.tif
A perspective view of the centrosymetric title complex, with atom numbering and non H-atoms represented as 40% probability displacement ellipsoids. Symmetry code (i): −*x*, −*y*, −*z* + 1.

b . DOI: 10.1107/S2056989015001292/zs2324fig2.tif
A packing diagram of the title complex viewed along the *b* axis with C—H⋯S inter­actions shown by dotted lines.

**Fig. 3.**
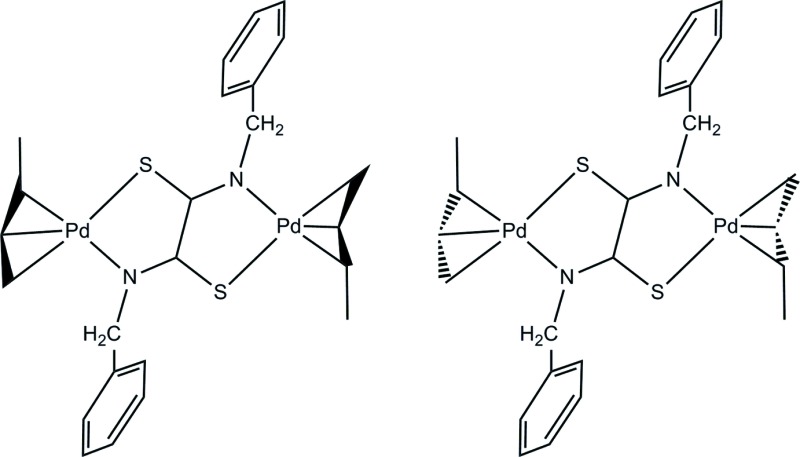
Enantiomeric symmetry-related C2-pairs in the title compound.

C . DOI: 10.1107/S2056989015001292/zs2324fig3.tif
Enanti­omeric symmetry-related *C*2-pairs in the title compound.

CCDC reference: 1044665

Additional supporting information:  crystallographic information; 3D view; checkCIF report

## supplementary crystallographic information

### S1. Comment

η^3^-Allyl palladium complexes are very widely studied because they can act as precursors or intermediates in different catalytic processes (Jalòn *et al.*, 2005). In the dimeric title η^3^-allyl–palladium(II) complex [Pd_2_(C_4_H_6_)_2_(S_2_N_2_C_16_H_14_)] with the dibenzyldithiooximidate (DTO) ligand, the asymmetric unit consists of a half molecule lying across an inversion center (Fig. 1). The DTO ligands adopt a binucleating role through the S and N atoms bridging two equivalent palladium centres with Pd—N6 and Pd—S bond distances of 2.080 (7) Å and 2.3148 (9) Å, respectively. The C4—C4^i^ bond length within the DTO ligand [1.517 (5) Å] is typical of values found in planar *trans*-dithiooxamides. The benzyl groups are oriented so as to maximize the intramolecular interaction between a benzylic H-atom and a sulfur atom [C5—H5···S^i^, 3.005 (4) Å: C—H···S, 116°], resulting in a dihedral angle of 77.1 (2)° between the benzene rings and the metal complex plane. With the allyl ligand, the η^3^ asymmetric coordination as well as its orientation with respect to the palladium centers are mainly determined by steric hindrance between crotyl and benzyl fragments placing the latter on the opposite side of the methyl group. The Pd—C distances for the three C-atoms of the crotyl ligand (C1, C2, C3) are 2.147 (4), 2.079 (5) and 2.098 (5) Å, respectively. Although the secondary dithiooxamide is chelating the metal *via* the N···S sites in this complex, structural parameters around the Pd^II^ are in good agreement with those in similar compounds in which the planar DTO bridge is chelating the "hard" palladium(II) *via**N,N'* atoms (Lanza *et al.*, 2000; Lanza *et al.*, 2003). The overall molecular packing, as shown in Fig. 2, no inter-complex hydrogen-bonding interactions are present.

The title compound consists of two halves each of which is made by two chiral planes: the one containing palladium and the other perpendicular to it, *i.e.* the η^3^-linked crotyl plane. The compound is a mesoform: in fact either chiral crotyl plane and palladium square plane of one half-molecule have opposite configurations with respect to the corresponding planes of the other half (Fig. 3). The compound might exist in different diastereomeric forms: one, represented here, is a mesoform having crotyl CH_3_*cis* to sulfur; the other is a racemate having crotyl CH_3_*cis* to sulfur and crotyl cusps oriented toward the same side of palladium molecular square planes. A more detailed description of the stereochemistry of the title compound has been done by us previously (Lanza *et al.*, 2000; Lanza *et al.*, 2011). Both the other mesoform and the racemate having the crotyl CH_3_*cis* to the nitrogen probably cannot be formed because of close contact between the crotyl CH_3_ and the DTO benzyl substituents.

### S2. Experimental

A solution of 1 mmol (365 mg) of [(η^3^crotyl)PdCl]_2_ 100 ml of chloroform was reacted with a 2 mmol equivalent of H_2_benzil_2_DTO. The solution turned orange and was left to stand for 30 min at room temperature. After the addition with stirring of 2 g of sodium bicarbonate, the mixture turned bright yellow. After filtration of the excess bicarbonate, the filtrate, which contained [(η^3^crotyl)Pd(H—*R*_2_—DTO κ-S,*S* Pd)], was reacted with 1 mmol (365 mg) of [(η^3^crotyl)PdCl]_2_. The mixture was refluxed for 24 h at 50 °C after which the solvent was removed, and the crude product was redissolved in a minimum amount of chloroform and loaded onto an alumina column equilibrated with petroleum ether. Elution with a petroleum ether/chloroform mixture (90:10) gave a yellow fraction from which the homobimetallic title complex [(η^3^crotyl)Pd]_2_(µ-benzyl_2_-DTO κ-N,*S* Pd, κ-N',*S*' Pd'] was crystallized. ^1^H NMR (300 MHz, CDCl_3_), δ (p.p.m.): 7.34 (m, 10H, N—CH_2_—C_6_H_5_), 4.86 (s, 4H, N—CH_2_—C_6_H_5_),3.96 (m, 2H, CH_2_CHCHCH_3_), 3.94 (d, 2H, CHsyn-antiCHCHCH_3_), 2.80 and 2.76 (2 d, 4H, CH*_syn_*H_anti_CHCHCH_3_), 1.76 (d, 6H, CH_2_CHCHCH_3_. ^13^C NMR (75 MHz, CDCl_3_), δ (p.p.m.):, 128.6–126.4 (Ph—CH), 114.2 (CH_3_—CH—CH_2_), 80.4 (CH_3_—CH—CH—CH_2_), 57.4, 57.5 (CH_3_—CHCH-CH_2_), 52.3 (N—CH_2_—C_6_H_5_), 18.2 (CH_3_—CH—CH—CH_2_).

### S3. Refinement

Although the hydrogen atoms could be clearly identified in a difference Fourier synthesis, all were idealized and refined at calculated positions riding on the carbon atoms with C–H distances of 0.96 Å (methyl), 0.97 Å (methylene), 0.93 Å (aromatic) and 0.97 Å for C1 and 0.98 Å for C2 and C3. Carbon atoms of the η^3^-linked crotyl fragment show large anisotropic displacement parameters giving rise to unusual C—C bond lengths and short H—H separations.

### Crystal data

**Table:**

[Pd_2_(C_4_H_7_)_2_(C_16_H_14_N_2_S_2_)]	*F*(000) = 1240
*M**_r_* = 621.40	*D*_x_ = 1.708 Mg m^−^^3^
Monoclinic, *C*2/*c*	Mo *K*α radiation, λ = 0.71073 Å
Hall symbol: -C 2yc	Cell parameters from 236 reflections
*a* = 18.3240 (2) Å	θ = 4.3–24.0°
*b* = 7.1660 (1) Å	µ = 1.67 mm^−^^1^
*c* = 19.5080 (2) Å	*T* = 298 K
β = 109.341 (4)°	Prismatic, yellow
*V* = 2417.03 (7) Å^3^	0.35 × 0.10 × 0.08 mm
*Z* = 4	

### Data collection

**Table:**

Bruker APEXII CCD diffractometer	2638 independent reflections
Radiation source: fine-focus sealed tube	2474 reflections with *I* > 2σ(*I*)
Graphite monochromator	*R*_int_ = 0.019
φ and ω scans	θ_max_ = 27.0°, θ_min_ = 2.7°
Absorption correction: multi-scan (*SADABS*; Bruker, 2012)	*h* = −23→23
*T*_min_ = 0.611, *T*_max_ = 0.746	*k* = −9→9
34315 measured reflections	*l* = −24→24

### Refinement

**Table:**

Refinement on *F*^2^	0 restraints
Least-squares matrix: full	Hydrogen site location: inferred from neighbouring sites
*R*[*F*^2^ > 2σ(*F*^2^)] = 0.031	H-atom parameters constrained
*wR*(*F*^2^) = 0.085	*w* = 1/[σ^2^(*F*_o_^2^) + (0.0388*P*)^2^ + 8.6756*P*] where *P* = (*F*_o_^2^ + 2*F*_c_^2^)/3
*S* = 1.03	(Δ/σ)_max_ = 0.001
2638 reflections	Δρ_max_ = 1.36 e Å^−^^3^
136 parameters	Δρ_min_ = −0.62 e Å^−^^3^

### Special details

**Table:**

Geometry. All e.s.d.'s (except the e.s.d. in the dihedral angle between two l.s. planes) are estimated using the full covariance matrix. The cell e.s.d.'s are taken into account individually in the estimation of e.s.d.'s in distances, angles and torsion angles; correlations between e.s.d.'s in cell parameters are only used when they are defined by crystal symmetry. An approximate (isotropic) treatment of cell e.s.d.'s is used for estimating e.s.d.'s involving l.s. planes.
Refinement. Refinement of *F*^2^ against ALL reflections. The weighted *R*-factor *wR* and goodness of fit *S* are based on *F*^2^, conventional *R*-factors *R* are based on *F*, with *F* set to zero for negative *F*^2^. The threshold expression of *F*^2^ > 2σ(*F*^2^) is used only for calculating *R*-factors(gt) *etc*. and is not relevant to the choice of reflections for refinement. *R*-factors based on *F*^2^ are statistically about twice as large as those based on *F*, and *R*- factors based on ALL data will be even larger.

### Fractional atomic coordinates and isotropic or equivalent isotropic displacement parameters (Å^2^)

**Table:**

	*x*	*y*	*z*	*U*_iso_*/*U*_eq_	
Pd1	0.01936 (2)	0.33811 (4)	0.42230 (2)	0.04724 (11)	
S	0.10566 (5)	0.15413 (15)	0.50967 (6)	0.0622 (3)	
N6	−0.06138 (15)	0.1444 (4)	0.43152 (13)	0.0420 (6)	
C5	−0.14245 (18)	0.1654 (5)	0.38465 (17)	0.0483 (8)	
H2A	−0.16	0.2904	0.3902	0.058*	
H2B	−0.1743	0.0779	0.4001	0.058*	
C4	0.04236 (16)	−0.0084 (4)	0.52284 (14)	0.0381 (6)	
C6	−0.15288 (17)	0.1315 (4)	0.30539 (16)	0.0401 (6)	
C8	−0.2302 (3)	0.1740 (6)	0.1805 (2)	0.0663 (11)	
H5	−0.2741	0.2225	0.1459	0.08*	
C7	−0.2179 (2)	0.2040 (5)	0.25323 (19)	0.0528 (8)	
H6	−0.2534	0.2732	0.2674	0.063*	
C10	−0.1143 (2)	−0.0012 (7)	0.2104 (2)	0.0705 (11)	
H7	−0.0792	−0.0712	0.1959	0.085*	
C11	−0.10169 (19)	0.0268 (6)	0.28338 (19)	0.0540 (8)	
H8	−0.0585	−0.0253	0.3178	0.065*	
C9	−0.1782 (3)	0.0736 (7)	0.1589 (2)	0.0710 (12)	
H9	−0.1861	0.056	0.1098	0.085*	
C12	0.1701 (3)	0.5766 (8)	0.4400 (3)	0.0902 (15)	
H12A	0.1966	0.4599	0.4537	0.135*	
H12B	0.1764	0.6502	0.4827	0.135*	
H12C	0.1914	0.6426	0.4082	0.135*	
C1	−0.0440 (3)	0.5326 (7)	0.3405 (3)	0.0850 (15)	
H1A	−0.061	0.4886	0.2906	0.102*	
H1B	−0.0818	0.6099	0.352	0.102*	
C2	0.0273 (3)	0.5825 (8)	0.3678 (4)	0.103 (2)	
H2	0.0183	0.6645	0.4044	0.124*	
C3	0.0927 (4)	0.5440 (11)	0.4048 (5)	0.157 (4)	
H3	0.1024	0.4731	0.3657	0.188*	

### Atomic displacement parameters (Å^2^)

**Table:**

	*U*^11^	*U*^22^	*U*^33^	*U*^12^	*U*^13^	*U*^23^
Pd1	0.04780 (16)	0.05170 (18)	0.04023 (15)	−0.00269 (11)	0.01192 (11)	0.00890 (10)
S	0.0387 (4)	0.0756 (7)	0.0600 (5)	−0.0124 (4)	−0.0002 (4)	0.0248 (5)
N6	0.0365 (12)	0.0535 (15)	0.0314 (12)	0.0015 (11)	0.0049 (10)	0.0057 (11)
C5	0.0354 (15)	0.068 (2)	0.0374 (15)	0.0097 (14)	0.0068 (12)	0.0095 (14)
C4	0.0359 (14)	0.0492 (16)	0.0258 (12)	−0.0023 (12)	0.0059 (11)	0.0012 (11)
C6	0.0338 (13)	0.0438 (16)	0.0371 (14)	0.0010 (12)	0.0044 (11)	0.0067 (12)
C8	0.069 (2)	0.067 (3)	0.0437 (19)	0.0010 (19)	−0.0071 (18)	0.0086 (17)
C7	0.0477 (17)	0.055 (2)	0.0462 (17)	0.0130 (15)	0.0021 (14)	0.0062 (15)
C10	0.061 (2)	0.090 (3)	0.064 (2)	−0.003 (2)	0.0254 (19)	−0.021 (2)
C11	0.0390 (15)	0.066 (2)	0.0502 (18)	0.0054 (15)	0.0054 (13)	−0.0051 (16)
C9	0.084 (3)	0.087 (3)	0.0376 (17)	−0.022 (2)	0.0136 (18)	−0.0077 (19)
C12	0.073 (3)	0.088 (4)	0.109 (4)	−0.022 (3)	0.028 (3)	0.004 (3)
C1	0.080 (3)	0.075 (3)	0.087 (3)	0.002 (2)	0.012 (2)	0.044 (3)
C2	0.078 (3)	0.084 (4)	0.134 (5)	−0.006 (3)	0.017 (3)	0.064 (4)
C3	0.078 (4)	0.145 (6)	0.206 (8)	−0.040 (4)	−0.009 (4)	0.128 (6)

### Geometric parameters (Å, º)

**Table:**

Pd1—C2	2.079 (5)	C8—H5	0.93
Pd1—N6	2.080 (3)	C7—H6	0.93
Pd1—C3	2.098 (5)	C10—C9	1.374 (6)
Pd1—C1	2.147 (4)	C10—C11	1.380 (5)
Pd1—S	2.3148 (9)	C10—H7	0.93
S—C4	1.722 (3)	C11—H8	0.93
N6—C4^i^	1.288 (4)	C9—H9	0.93
N6—C5	1.472 (4)	C12—C3	1.377 (7)
C5—C6	1.513 (4)	C12—H12A	0.96
C5—H2A	0.97	C12—H12B	0.96
C5—H2B	0.97	C12—H12C	0.96
C4—N6^i^	1.288 (4)	C1—C2	1.289 (7)
C4—C4^i^	1.517 (5)	C1—H1A	0.97
C6—C11	1.376 (5)	C1—H1B	0.97
C6—C7	1.386 (4)	C2—C3	1.208 (7)
C8—C9	1.367 (7)	C2—H2	0.98
C8—C7	1.378 (5)	C3—H3	0.98
			
C2—Pd1—N6	141.18 (17)	C9—C10—H7	119.7
C2—Pd1—C3	33.6 (2)	C11—C10—H7	119.7
N6—Pd1—C3	174.70 (17)	C6—C11—C10	120.2 (3)
C2—Pd1—C1	35.46 (19)	C6—C11—H8	119.9
N6—Pd1—C1	105.80 (15)	C10—C11—H8	119.9
C3—Pd1—C1	68.9 (2)	C8—C9—C10	119.5 (4)
C2—Pd1—S	135.22 (16)	C8—C9—H9	120.3
N6—Pd1—S	83.58 (7)	C10—C9—H9	120.3
C3—Pd1—S	101.66 (16)	C3—C12—H12A	109.5
C1—Pd1—S	170.53 (14)	C3—C12—H12B	109.5
C4—S—Pd1	99.53 (10)	H12A—C12—H12B	109.5
C4^i^—N6—C5	119.6 (3)	C3—C12—H12C	109.5
C4^i^—N6—Pd1	121.8 (2)	H12A—C12—H12C	109.5
C5—N6—Pd1	118.6 (2)	H12B—C12—H12C	109.5
N6—C5—C6	112.2 (3)	C2—C1—Pd1	69.4 (3)
N6—C5—H2A	109.2	C2—C1—H1A	116.7
C6—C5—H2A	109.2	Pd1—C1—H1A	116.7
N6—C5—H2B	109.2	C2—C1—H1B	116.7
C6—C5—H2B	109.2	Pd1—C1—H1B	116.7
H2A—C5—H2B	107.9	H1A—C1—H1B	113.7
N6^i^—C4—C4^i^	117.2 (3)	C3—C2—C1	148.5 (5)
N6^i^—C4—S	125.0 (2)	C3—C2—Pd1	74.1 (3)
C4^i^—C4—S	117.8 (3)	C1—C2—Pd1	75.2 (3)
C11—C6—C7	119.0 (3)	C3—C2—H2	94.3
C11—C6—C5	122.5 (3)	C1—C2—H2	94.3
C7—C6—C5	118.5 (3)	Pd1—C2—H2	94.3
C9—C8—C7	120.5 (4)	C2—C3—C12	156.0 (6)
C9—C8—H5	119.8	C2—C3—Pd1	72.3 (3)
C7—C8—H5	119.8	C12—C3—Pd1	130.4 (4)
C8—C7—C6	120.3 (4)	C2—C3—H3	93.1
C8—C7—H6	119.9	C12—C3—H3	93.1
C6—C7—H6	119.9	Pd1—C3—H3	93.1
C9—C10—C11	120.5 (4)		
			
C4^i^—N6—C5—C6	−112.8 (3)	C7—C6—C11—C10	1.6 (6)
Pd1—N6—C5—C6	67.6 (3)	C5—C6—C11—C10	179.4 (4)
Pd1—S—C4—N6^i^	−178.6 (3)	C9—C10—C11—C6	−0.6 (7)
Pd1—S—C4—C4^i^	1.6 (3)	C7—C8—C9—C10	1.4 (7)
N6—C5—C6—C11	23.2 (5)	C11—C10—C9—C8	−1.0 (7)
N6—C5—C6—C7	−159.0 (3)	Pd1—C1—C2—C3	12.2 (17)
C9—C8—C7—C6	−0.4 (6)	C1—C2—C3—C12	−175.4 (17)
C11—C6—C7—C8	−1.1 (6)	Pd1—C2—C3—C12	−163 (3)
C5—C6—C7—C8	−179.1 (4)	C1—C2—C3—Pd1	−12.3 (17)

Symmetry code: (i) −*x*, −*y*, −*z*+1.
