# Supplementary figures and images for: Crystal structure of (μ-N,N′-di­benzyl­dithio­oxamidato-κN,S:N′,S′)bis­[(η3-crotyl)palladium(II)]
Source: Acta Crystallogr E Crystallogr Commun. 2015 Jan 28;71(Pt 2):m40–1. doi: 10.1107/S2056989015001292 (PMC4384546; doi:10.1107/S2056989015001292)

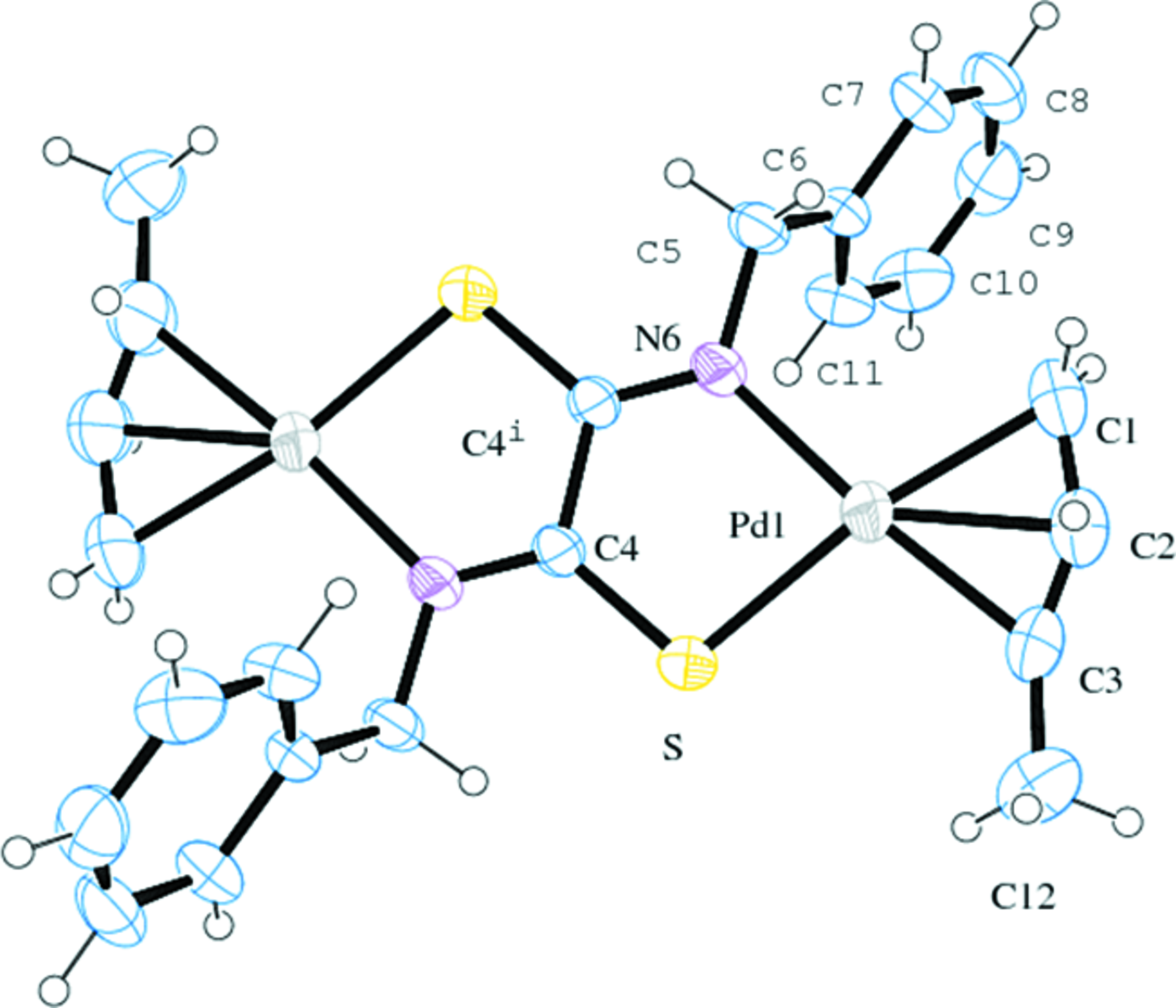

Supplement: Supplementary file 3 [file e-71-00m40-fig1.tif]

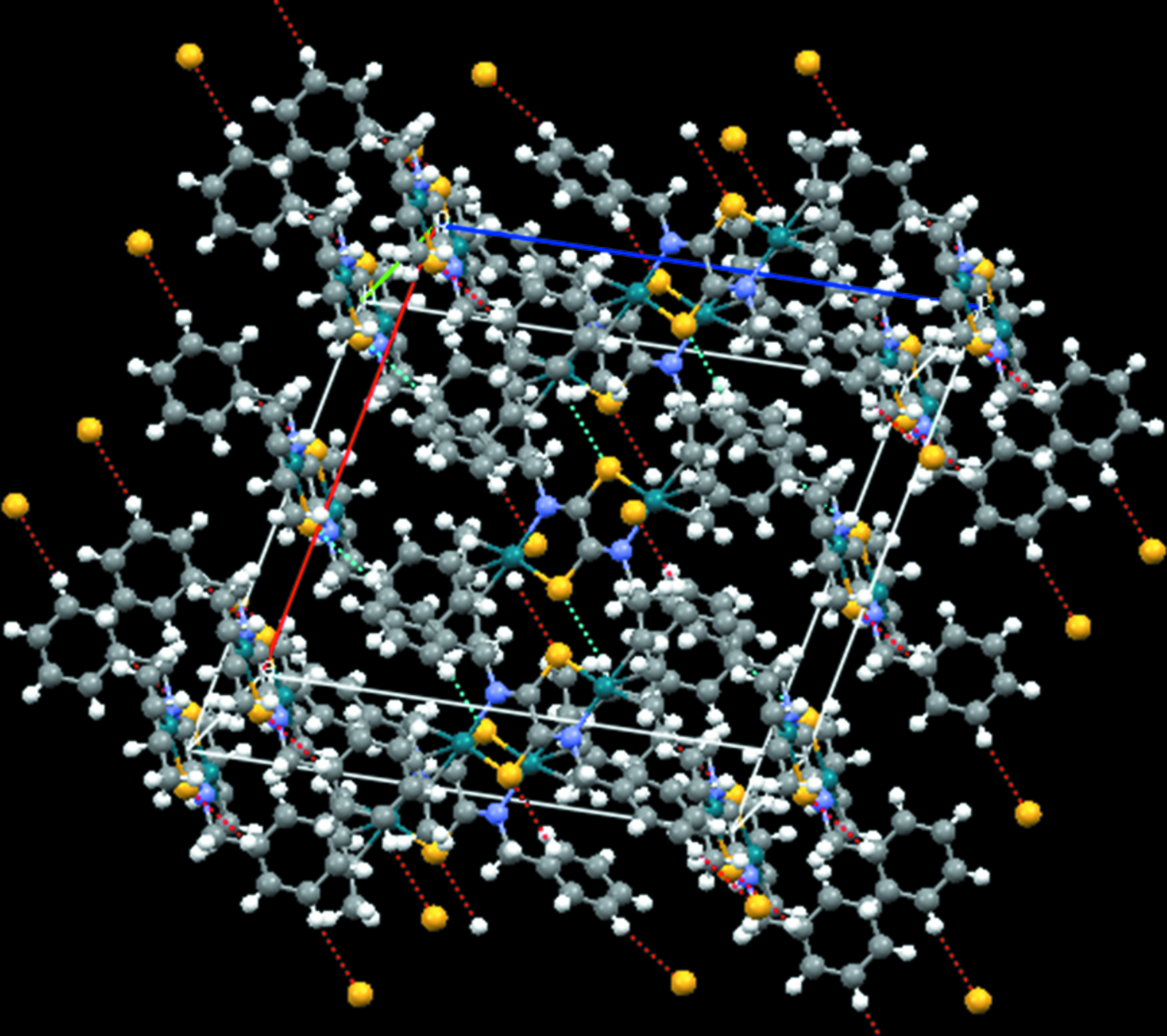

Supplement: Supplementary file 4 [file e-71-00m40-fig2.tif]

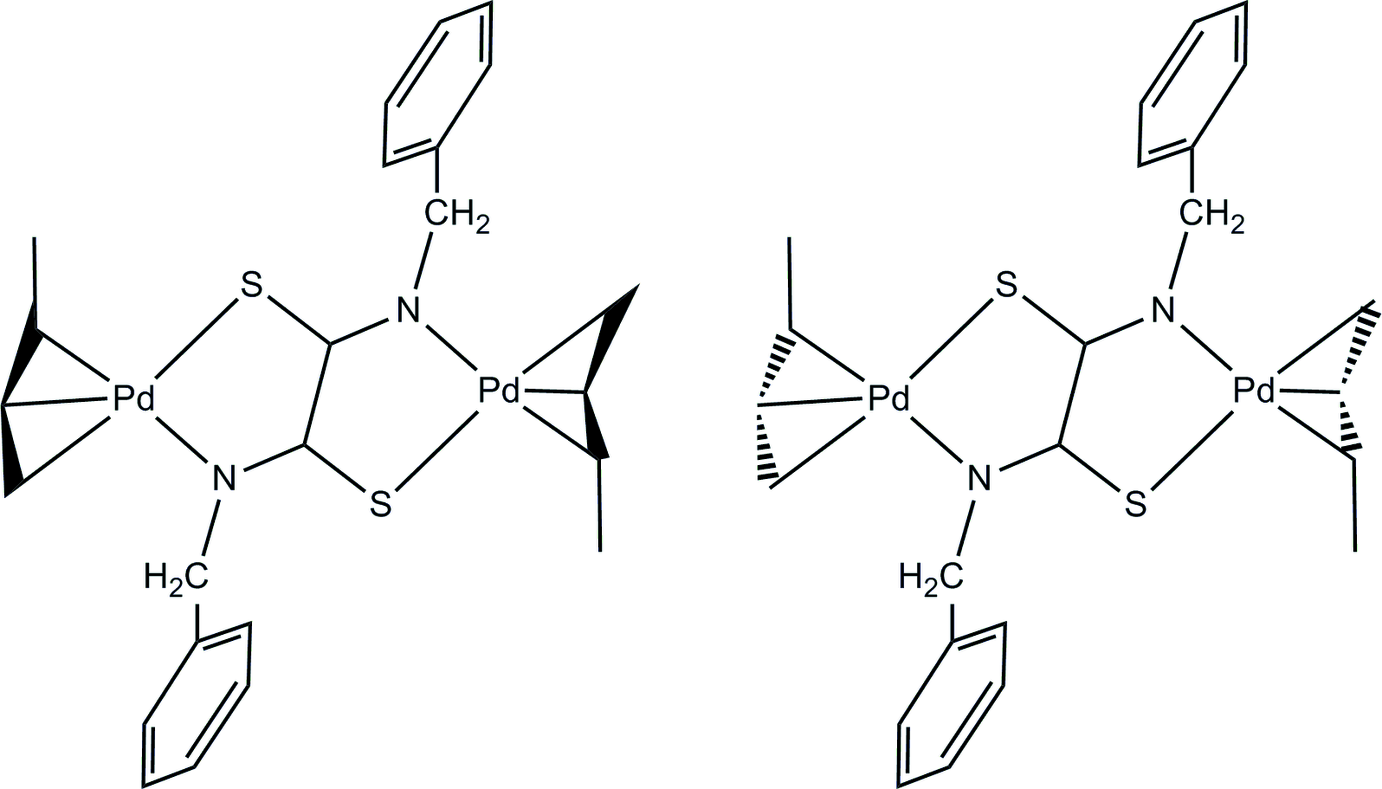

Supplement: Supplementary file 5 [file e-71-00m40-fig3.tif]
